# Acid sphingomyelinase mediates ferroptosis induced by high glucose via autophagic degradation of GPX4 in type 2 diabetic osteoporosis

**DOI:** 10.1186/s10020-023-00724-4

**Published:** 2023-09-14

**Authors:** Yun-xia Du, Yan-tao Zhao, Yong-xin Sun, Ai-hua Xu

**Affiliations:** 1 Department of Rehabilitation Medicine, The Second Hospital of Dalian Medical University, Dalian, Liaoning People’s Republic of China; 2 Department of Joint Surgery, Dalian Municipal Central Hospital Affiliated to Dalian University of Technology, Dalian, Liaoning People’s Republic of China; 3 Department of Rehabilitation Medicine, The First Affiliated Hospital of China Medical University, Shenyang, Liaoning People’s Republic of China

**Keywords:** Acid sphingomyelinase, Ceramide, Autophagy, Ferroptosis, Type 2 diabetic osteoporosis

## Abstract

**Background:**

Ferroptosis has been implicated in the pathological process of type 2 diabetic osteoporosis (T2DOP), although the specific underlying mechanisms remain largely unknown. This study aimed to clarify the role and possible mechanism of acid sphingomyelinase (ASM)-mediated osteoblast ferroptosis in T2DOP.

**Methods:**

We treated hFob1.19 cells with normal glucose (NG) and different concentrations of high glucose (HG, 26.25 mM, 35 mM, or 43.75 mM) for 48 h. We then measured cell viability and osteogenic function, quantified ferroptosis and autophagy levels, and measured the levels of ASM and ceramide in the cells. To further investigate the specific mechanism, we examined these indicators by knocking down ASM expression, hydroxychloroquine (HCQ) treatment, or *N*-acetylcysteine (NAC) treatment. Moreover, a T2DOP rat model was induced and microcomputed tomography was used to observe the bone microstructure. We also evaluated the serum levels of iron metabolism-associated factors, ceramide and lipid peroxidation (LPO) and measured the expression of ASM, LC3 and GPX4 in bone tissues.

**Results:**

HG inhibited the viability and osteogenic function of osteoblasts by inducing ferroptosis in a concentration-dependent manner. Furthermore, the expression of ASM and ceramide and autophagy levels were increased by HG treatment, and these factors were required for the HG-induced reactive oxygen species (ROS) generation and LPO. Similarly, inhibiting intracellular ROS also reduced HG-induced ASM activation and autophagy. ASM-mediated activation of autophagy was crucial for HG-induced degradation of GPX4, and inhibiting ASM improved osteogenic function by decreasing HG-induced autophagy, GPX4 degradation, LPO and subsequent ferroptosis. We also found that inhibiting ASM could alleviated ferroptosis and autophagy and improved osteogenic function in a T2DOP rat model.

**Conclusion:**

ASM-mediated autophagy activation induces osteoblast ferroptosis under HG conditions through the degradation of GPX4, providing a novel mechanistic insight into the treatment and prevention of T2DOP.

## Background

As a common complication of type 2 diabetes mellitus (T2DM), osteoporosis is characterized by reduced bone mass, augmented bone fragility and increased risk of fracture, which physically and economically affects the elderly population (Si et al. 2020). The occurrence of osteoporosis is mainly due to a decline in the osteoblastic function of osteoblasts and the hyperfunction of osteoclasts, which disrupts the balance between bone formation and bone absorption (Marini et al. 2021). At present, studies on the mode of osteoblast death have included ferroptosis, apoptosis, autophagy and oxidative stress (Wang et al. 2022; Suzuki et al. 2020; Shen et al. 2018; Li et al. 2021). Type 2 diabetic osteoporosis (T2DOP) involves complex pathological factors, and the mechanism has not been fully studied. Therefore, it is critical to study the pathogenesis of T2DOP and provide new directions for its prevention and treatment.

Ferroptosis is a new form of regulated cell death that was first reported in 2012, and mainly depends on iron accumulation and lipid peroxidation (LPO) (Dixon et al. 2012). Ferroptosis has been widely reported to be involved in the pathogenesis of many diseases, including nervous system diseases, tumors, ischemia–reperfusion injury and metabolic diseases (Li et al. 2019, 2020; Ajoolabady et al. 2021; Yan et al. 2020). Many studies have shown that osteoporosis is closely related to iron overload, which leads to bone loss and changes in bone microstructure and biomechanics by disrupting the balance between bone formation and bone destruction, thereby causing osteoporosis (Jiang et al. 2022). In recent years, studies have confirmed that osteoblast ferroptosis is involved in the pathological process of T2DOP, and inhibiting osteoblast ferroptosis is expected to become a new and effective treatment for T2DOP (Ma et al. 2020). However, the specific pathways associated with osteoblast ferroptosis have not been fully elucidated.

Although the initial study showed that ferroptosis was distinct from autophagy and other types of cell death, recent studies have demonstrated that the activation of ferroptosis is dependent on the induction of autophagy (Liu et al. 2020; Zhou et al. 2019). It has been reported that ferritin autophagy, lipid autophagy, clockophagy and chaperone-mediated autophagy play an important roles in ferroptosis (Santana-Codina et al. 2021; Bai et al. 2019; Yang et al. 2019; Chen et al. 2021). Therefore, exploring the relationship between autophagy and ferroptosis will not only reveal the mechanism of this type of regulated cell death, but also provide new therapeutic targets for ferroptosis-related diseases.

Acid sphingomyelinase (ASM) is a key enzyme in sphingolipid metabolism that can catalyze the production of ceramide from sphingomyelin. The ASM/ceramide system can regulate a variety of signaling pathways, including apoptosis, autophagy, differentiation and migration, and is widely involved in the pathogenesis of many diseases, including diabetes, ischemia–reperfusion injury, nervous system diseases, and cardiovascular diseases (Liu et al. 2023). Some studies have shown that the level of ASM is increased in the plasma of T2DM patients, which inhibits the repair potential of mature retinal endothelial cells and circulating angiogenic cells, and inhibiting ASM activity can significantly improve the level of type 2 diabetes retinopathy (Ensari et al. 2021). However, the role of ASM in T2DOP has not been reported. A recent study showed that ASM-mediated autophagy played an important role in the ferroptosis in HT-1080 cells induced by the ferroptosis-inducer erastin, providing a new pathway for the regulation of ferroptosis (Thayyullathil et al. 2021).

Therefore, this study explored the role of ASM-mediated and autophagy-dependent ferroptosis in osteoblasts in T2DOP and clarified the specific regulatory mechanism, aiming to provide a new target for the prevention and treatment of T2DOP.

## Methods

### Cell culture conditions and treatments

The human osteoblast cell line hFob1.19 was obtained from the Cell Bank of the Chinese Academy of Sciences (Shanghai, China). The cells were cultured in DMEM/F-12 containing 10% FBS in a humidified 5% CO_2_ atmosphere at 37 °C (Thermo Scientific, USA). hFob1.19 cells were first cultured in (NG) normal glucose (NG, 17.5 mM) and different concentrations of high glucose (HG, 26.25 mM; 35 mM; 43.75 mM) for 48 h. Then, we chose an optimal glucose concentration as the HG condition for further experiments. To investigate the presence and potential mechanism of ferroptosis in hFob1.19 cells, the ferroptosis inhibitor ferrostatin-1 (Fer-1) (5 µM), autophagy inhibitor hydroxychloroquine (HCQ, 10 µM) and *N*-acetylcysteine (NAC, 5 mM) were added to the cell cultures for 12 h.

### shRNA-mediated knockdown of ASM, Beclin 1 and Atg5

The expression of ASM, Beclin 1 and Atg5 was knocked down by transfecting hFob1.19 cells with shRNAs purchased from GeneChem Corporation (Shanghai, China). Briefly, 1 × 10^6^ cells were seeded in 6-well plates the day before transfection, and 40 µl of transfection reagent (Beyotime Biotechnology, Shanghai, China) and lentiviral particles (20 multiplicity of infection, MOI) were added to the culture on the 2nd day. After 72 h of transfection, hFob1.19 cells were treated as indicated.

### Cell viability analysis

hFob1.19 cells (5000 cells/well) were seeded in 96-well plates and were subjected to the different treatments. Cell viability was evaluated using the Cell Counting Kit-8 (CCK8, Beyotime Biotechnology) according to the manufacturer’s instructions, and the optical density (OD) values of the cells were measured at 450 nm using a microplate reader (Bio-Tek Instruments, Winooski, VT, USA). In addition, we assessed cell proliferation using an EdU assay kit (RiboBio, Guangzhou, China) according to the manufacturer’s instructions.

### Detection of cytotoxicity

The cytotoxicity of hFob1.19 cells was determined using an LDH cytotoxicity assay kit (Beyotime Biotechnology). The cells were lysed and incubated with 60 µl of LDH working solution at 25 °C for 30 min in the dark. Then, the OD values of the cells were measured using a microplate reader (Bio-Tek Instruments, Winooski, VT, USA) at 490 nm .

### Intracellular ROS measurement

ROS production was detected using an ROS-sensitive fluorescent indicator DHE assay kit (Beyotime Biotechnology). ROS generation was represented by total DHE fluorescence, and the fluorescence intensity was detected using a laser scanning confocal microscope (LSCM, Olympus, Tokyo, Japan).

### Malondialdehyde (MDA) and 4-HNE measurement to determine LPO

MDA levels in cells were measured using an LPO MDA assay kit (Beyotime Biotechnology). Cellular proteins were extracted and added to MDA detection working solution and then heated in boiling water for 15 min. The absorbance was measured at 532 nm using a microplate reader to calculate the MDA levels. 4-HNE levels were determined using a 4-HNE assay kit (Abcam Inc., Cambridge, MA, USA) according to the manufacturer’s instructions, and the OD values of the cells were determined using a microplate reader (Bio-Tek Instruments, Winooski, VT, USA) at 450 nm. Then, a standard curve was drawn based on the OD values of the standard, and the concentration of the samples was calculated.

#### Fluorescence probe detection to determine LPO

To evaluate LPO, the cells were incubated with C11 BODIPY 581/591 (Shanghai Mao Kang Biotechnology Co, Ltd, Shanghai, China) (2 µM in HEPES-buffered HBSS) for 30 min at 37 °C in the dark, and the green and red fluorescence images were acquired simultaneously using double wavelength excitation (laserlines; 488 and 565 nm) and detection (emission bandpass filters 530/30 and 590/30) under an LSCM (Olympus, Tokyo, Japan).

### Measurement of intracellular Fe^2+^

FeRhonox-1 (Shanghai Mao Kang Biotechnology Co, Ltd), a commercial fluorescent probe that specifically binds to Fe^2+^, was used to determine intracellular Fe^2+^ levels. hFob1.19 cells were incubated with 5 µM FeRhoNox-1 dye solution for 60 min at 37 °C after which the fluorescence intensity was measured at an excitation wavelength of 532 nm and an emission wavelength of 570 nm using an LSCM (Olympus, Tokyo, Japan). Moreover, an iron assay kit (Abcam, Cambridge, MA) was subsequently used to quantify total iron levels in the cell lysates according to the manufacturer’s instructions, and spectrophotometry was used to measure absorbance at a wavelength of 593 nm.

### Alkaline phosphatase (ALP) staining

The osteogenic function of hFob1.19 cells was evaluated using an ALP staining kit (Beyotime Biotechnology). Briefly, the cells were cultured in a 24-well plate, and immediately after the culture media was removed, PBS was added to gently rinse the cells. After being fixed with prechilled fixative on ice for 10 min, the cells were permeabilized and incubated for 1 min at − 20 °C. Then, ALP substrate was added to each well and incubated at 37 °C for 45 min. Finally, nuclear stain solution was added to the cells, and after 10 min of staining, the cells were visualized using a light microscope (Nikon, Japan). Blue areas indicated ALP activity.

### Alizarin Red S (ARS) staining

To measure bone nodule formation after osteogenic differentiation, extracellular matrix calcium deposits were evaluated using ARS staining. hFob1.19 cells were inoculated at a density of 2 × 10^5^/well under different conditions, followed by 2 weeks of culture in osteogenic medium. After routine washing and fixation, the cells were stained with 40 mM ARS solution (Sigma-Aldrich, Germany) for 5 min. Then, images were obtained by phase-contrast microscopy (Nikon, Japan), and the density of calcium nodules was assessed by Image-Pro Plus 6.0.

### Ceramide measurement

High performance liquid chromatography (HPLC) was used for the determination of ceramide in hFob1.19 cells, as described previously (Thayyullathil et al. 2021). Ceramide levels in serum were measured using a ceramide assay kit (Shanghai Yingxin Laboratory Equipment Co., Ltd, China). The absorbance was measured at 450 nm using a microplate reader to calculate the ceramide level according to the instructions.

### Western blot analysis

After the treatments, hFob1.19 cells were washed with PBS and lysed with RIPA buffer for 30 min. The samples was centrifuged at 12,000×*g* at 4 °C for 30 min, and the supernatant containing the total protein was harvested. We measured and normalized the protein concentration of each sample by the bicinchoninic acid (BCA) method. Approximately 30 µg of protein was separated by 10% sodium dodecyl sulfate‒polyacrylamide gel electrophoresis (SDS‒PAGE) at 80 V and then transferred to PVDF membranes (General Electric Company, Madison, USA) at 110 mA for 60 min. The membranes were blocked with 5% (w/v) skim milk for 1 h followed by incubation with the corresponding primary antibodies for 12 h at 4 °C. After the membranes were washed three times with TBST, we incubated the membranes with secondary antibodies (Cell Signaling Technology, Danvers, USA) (at a dilution ratio of 1:4000) for 2 h. The protein bands were visualized using the EC3 Imaging System (UVP Inc. Upland, CA, USA), and the relative in density of each band was quantified using the ImageJ software (National Institutes of Health, Bethesda, MD, USA). The following primary antibodies were used: ASM (rabbit, dilution of 1:200, ab272729, Abcam, Cambridge, UK), GPX4 (rabbit, dilution of 1:200, ab125066, Abcam), Beclin 1 (rabbit, dilution of 1:200, ab207612, Abcam), Atg5 (rabbit, dilution of 1:200, ab108327, Abcam), LC3II (rabbit, dilution of 1:200, ab192890, Abcam), and β-actin (rabbit, ab115777, Abcam), which were all purchased from Abcam (Cambridge, UK).

### Determination of autophagic flux

The tandem fluorescent protein expression vector mRFP-GFP-LC3 (ptfLC3) (Hanbio Biotechnology Co., Ltd, Shanghai, China) was used to monitor autophagic flux based on the different pH stabilities of the fluorescent proteins mRFP and GFP. After the different treatments, hFob1.19 cells were transfected with ptfLC3, and fluorescence images were acquired using an LSCM. Autophagic flux was determined by quantifying the number of green puncta and red puncta in cells, and at least 20 cells were counted in triplicate per condition. Green puncta mainly represent autophagosomes, whereas red puncta represent both autophagosomes and autolysosomes in individual images. Green puncta were overlaid with red puncta and appeared as yellow puncta in the merged images, indicating autophagosomes, and free red puncta in the merged images indicated autolysosomes.

### Experimental animals

A total of sixty specific-pathogen-free Sprague Dawley (SD) rats weighing 200 ± 20 g were purchased from the Experimental Animals Department of China Medical University. Thirty rats were used as controls, while the other thirty rats were used to establish the T2DOP model. The model rats were fed with a high-fat diet for 2 months combined with a small dose of streptozotocin (30 mg/kg) (S0130, Sigma-Aldrich, St. Louis, MO, USA) that was intraperitoneally injected. After 72 h, the type 2 diabetes model was successfully established when the fasting blood glucose (FBG) exceeded 7.8 mmol/l and the insulin sensitivity index (ISI) decreased. Then, the rats were maintained for 2 months to induce osteoporosis. At the beginning of the third month, the model rats were injected with 200 µl of ASM-siRNA (Ruibo Biological Technology, Guangzhou, China) through the tail vein every 2 days for 2 weeks to establish a model of ASM interference after T2DOP. After continued feeding for 2 weeks, the rats were sacrificed for subsequent experiments. Control rats were fed normal food and water under normal laboratory conditions and were injected with 200 µl of NC-siRNA after being fed for 3 months.

### Microcomputed tomography (Micro-CT) assessment

To measure bone microstructures, the rats were sacrificed when their skeleton matured at 12 weeks of age according to a previous study. After sacrificing the rats by cervical dislocation, the right femur was removed and assessed by micro-CT scanning. The scanning parameter settings were as follows: 1024 × 1024 image matrix, 80 kV voltage, 80 µA current and 2.96 s exposure time. Images were then recombined using micro-CT, and the following parameters were measured: bone mineral density (BMD), trabecular number (Tb.N), trabecular thickness (Tb.Th) and trabecular bone volume per tissue volume (BV/TV).

### Immunohistochemistry (IHC)

After fixation, decalcification, embedding and sectioning, the bone tissue sections were deparaffinized in xylene and rehydrated in a graded series of ethanol. After antigen retrieval, peroxidase activity was quenched in 3% H_2_O_2_ for 15 min. The sections were blocked with 10% goat serum, and then incubated with primary rabbit monoclonal anti-ASM (1:200; ab272729, Abcam), anti-GPX4 (1:200; ab125066, Abcam), anti-Beclin 1 (1:200; ab207612, Abcam) or anti-LC3II (1:200; ab192890, Abcam) overnight at 4 °C. The next day, secondary goat anti-rabbit antibodies (SAP-9100, ZsBio, Beijing, China) were added and incubated for 2 h at 37 °C. Then, the sections were processed with ABC working solution (Zsbio) for 25 min at 37 °C and incubated with 3,3-diaminobenzidine (DAB; Zsbio). After the samples were thoroughly washed, images were acquired using a microscope (Leica Microsystems). GPX4, ASM, Beclin 1, and LC3 expression was semiquantitatively analyzed using ImageJ software.

### Determination of serum iron ions and MDA levels

The corresponding kits were purchased from Nanjing Jiancheng Bioengineering Institute. Serum samples were collected from the rats, and the levels of iron ions and MDA were measured. According to the instructions, the OD values were measured at 520 and 532 nm, respectively.

### Determination of serum iron ions, MDA, ASM activity and ceramide levels

The corresponding kits were purchased from Nanjing Jiancheng Bioengineering Institute. Serum samples were collected from the rats, the levels of iron ions and MDA were measured, and the OD values were measured at 520 and 532 nm, according to the instructions. We used HPLC to determine the activity of ASM and the levels of ceramide in rat serum.

### Statistical analysis

Statistical analysis was performed using GraphPad Prism 9.0 software (GraphPad Software, Inc., La Jolla, CA, USA). Each experiment was repeated three times, and all quantitative variables are expressed as the mean ± standard deviation. Student’s *t* test was used to evaluate the differences between two groups and one-way ANOVA was used for comparisons among multiple groups. Differences were considered significant when P < 0.05.

## Results

### HG induced ferroptosis in hFob1.19 cells in a dose-dependent manner

**Fig. 1:**
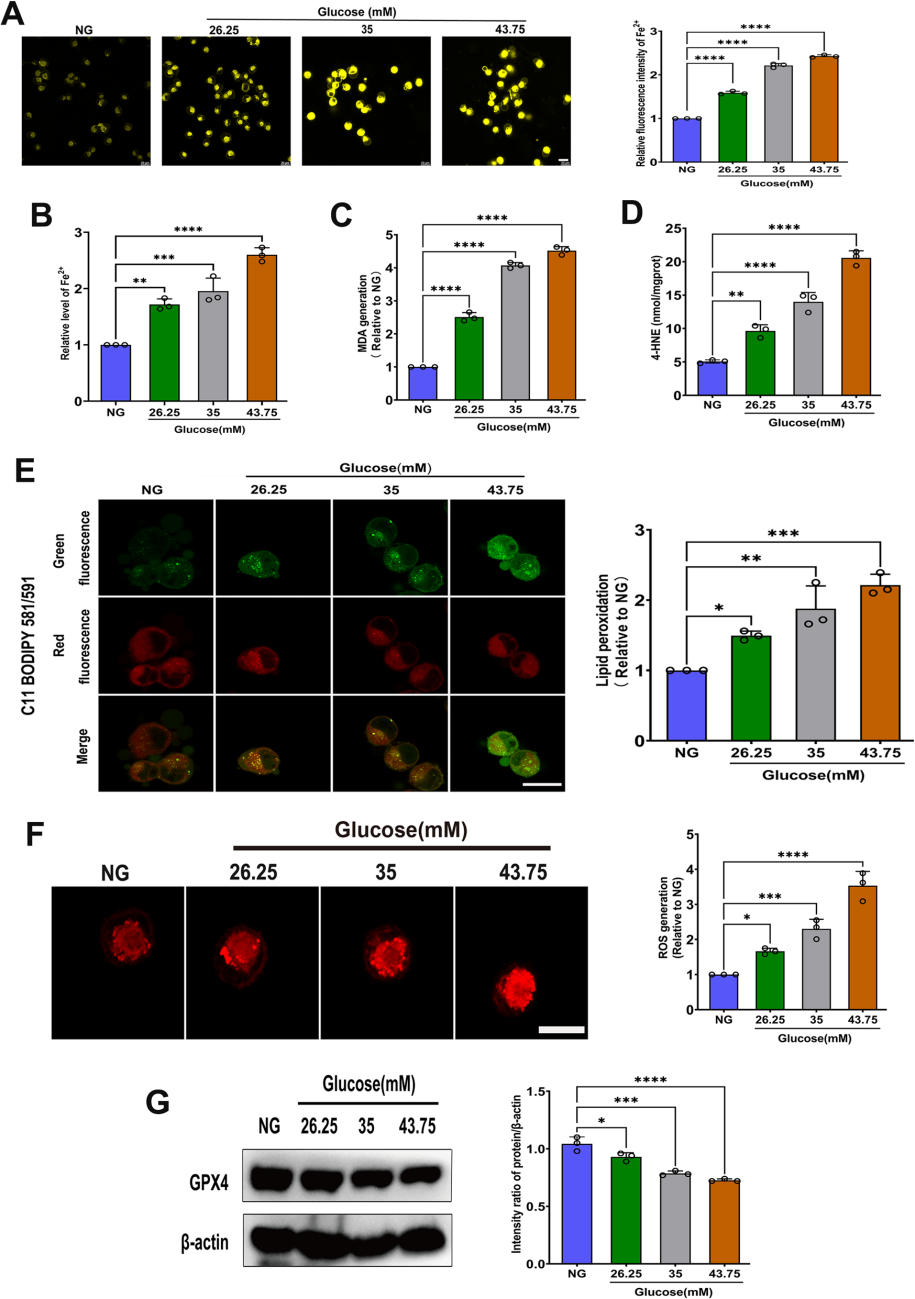
Ferroptosis was induced by HG in hFob1.19 cells. Cells were treated with NG (17.5 mM) and HG (26.25 mM, 35 mM, 43.75 mM, respectively) for 48 h. Following the treatments, **A** iron ions level was measured using the FeRhonox-1 fluorescent probe. Scale bar = 20 μm. **B** Intracellular iron levels were assessed using a commercial assay. **C** MDA assay was performed. **D** Lipid peroxidation was detected using the 4-HNE assay kits. **E** C11 BODIPY 581/591 fluorescent probe was used to detect LPO. Scale bar = 20 μm. **F** ROS generation was determined by DHE assay kits. Scale bar = 20 μm. **G** Western blot analysis was carried out to assay GPX4 expression. All data are presented as the mean ± SD of three independent experiments. *P < 0.05, **P < 0.01,***P < 0.001, ****P < 0.0001

Ferroptosis is a specific type of cell death defined by iron accumulation and LPO that involves ROS accumulation and decreased GPX4 activity. hFob1.19 cells were treated with different concentrations of high glucose (HG, 26.25 mM; 35 mM and 43.75 mM), and the normal glucose (NG) group was treated with 17.5 mM glucose. After 48 h of treatment with HG, we found an increased levels of intracellular iron ions (Fig. 1A, B). Moreover, lipid peroxides and ROS accumulated in HG-treated cells, as shown by MDA analysis (Fig. 1C), 4-HNE determination (Fig. 1D), fluorescent LPO probes (Fig. 1E), and DHE assay (Fig. 1F), and these effects were positively correlated with HG concentrations. GPX4, which is a marker of ferroptosis, was decreased by all concentrations of HG treatments (Fig. 1G). These data indicate that HG induces ferroptosis in osteoblasts in a dose-dependent manner.

### hFob1.19 cell viability and osteogenic function dose-dependently decreased in response to HG treatment

**Fig. 2:**
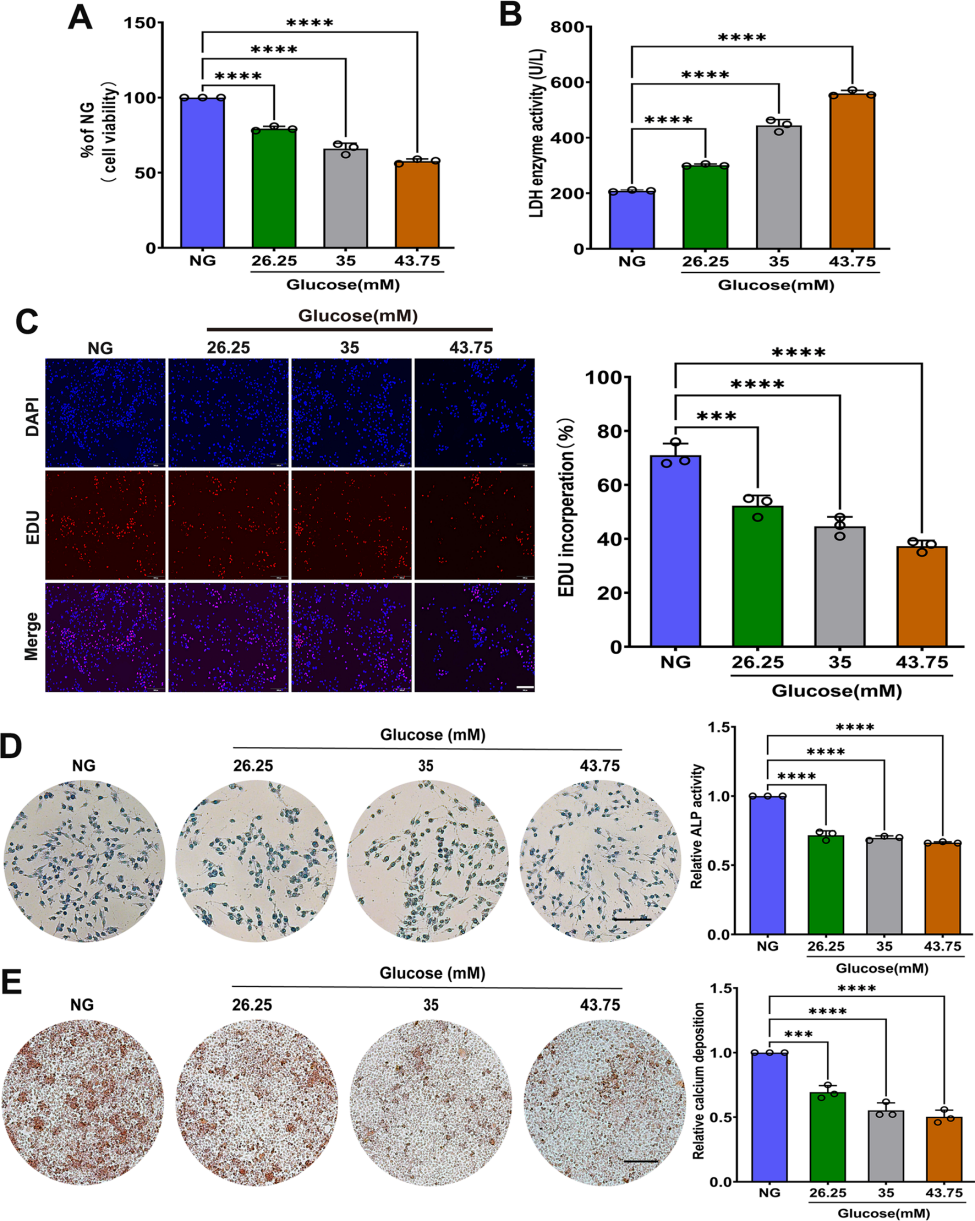
hFob1.19 cells viability and osteogenic function were detected. Cells were treated with NG (17.5 mM) and HG (26.25 mM, 35 mM, 43.75 mM, respectively) for 48 h. Following the treatment, **A** cell viability by CCK-8 was assessed and the data were expressed as values relative to the NG group. **B** The LDH enzyme activity was quantified using LDH content kit. **C** The proliferation of hFob1.19 cells detected by EdU assay. Scale bar = 200 μm. **D** ALP activity was determined after indicated treatments and the data were expressed as values relative to the NG group. **E** After osteogenic differentiation for 2 weeks, mineralized extracellular matrix in hFob1.19 cells was shown by alizarin red S staining, and the data were expressed as values relative to the NG group. Scale bar = 200 μm. All data are presented as the mean ± SD of three independent experiments. ***P < 0.001, ****P < 0.0001

Treatment with HG reduced hFob1.19 cell viability in a dose-dependent manner after 48 h. As shown in Fig. 2A, cell viability rates were 79.3%, 66%, and 57.7% in hFob1.19 cells following 48 h of treatment with 26.25 mM, 35 mM, and 43.75 mM glucose, respectively. To further examine cytotoxicity, we measured LDH activity in the medium, which increased with increasing HG concentrations (Fig. 2B). Moreover, the EdU assay showed that HG reduced cell proliferation in a dose-dependent manner (Fig. 2C). Next, ALP staining and ARS staining were used to evaluate the osteogenic function of hFob1.19 cells. The results showed that HG dose-dependently reduced ALP activity (Fig. 2D) and mineralized nodule formation (Fig. 2E) in hFob1.19 cells, suggesting that HG was negatively correlated with osteogenic ability. These results demonstrate that hFob1.19 cell viability and osteogenic function dose-dependently decrease in response to HG treatment, and we chose 35 mM glucose as the HG condition for further experiments.

### Autophagy levels and ASM/ceramide expression increased in hFob1.19 cells by HG treatment

**Fig. 3:**
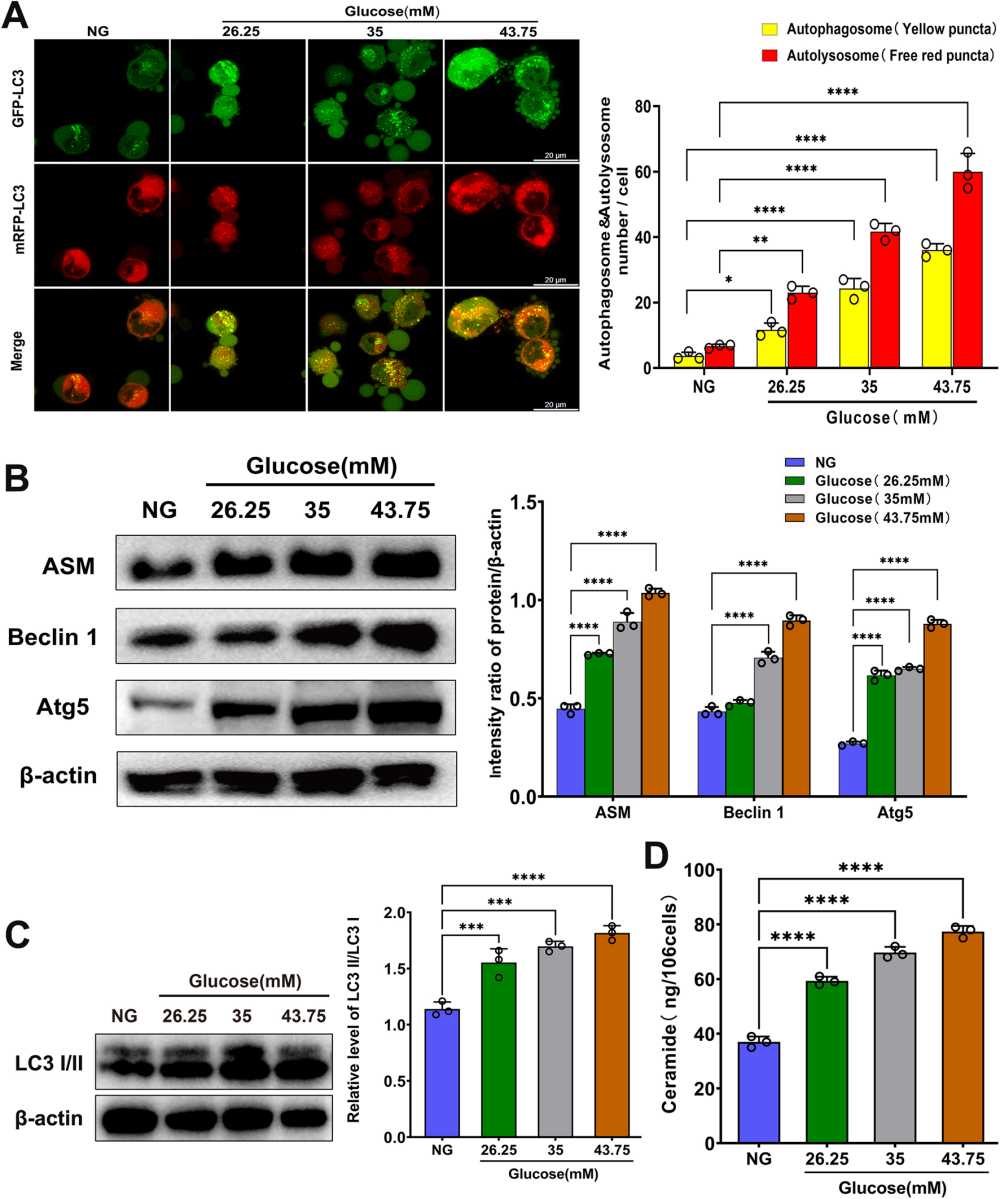
Autophagy level and ASM/ceramide system expression were measured in hFob1.19 cells. Cells were treated with NG (17.5 mM) and HG (26.25 mM, 35 mM, 43.75 mM, respectively) for 48 h. **A** hFob1.19 cells expressing ptfLC3 were treated with different conditions, and fluorescent images were captured using LSCM. The number of autophagosomes represented by yellow puncta and autolysosomes represented by red puncta in merged images. Scale bar = 20 μm. **B** Western blot analysis of indicated proteins were analyzed. **C** Western blot analysis of LC3 II was carried out. **D** Ceramide levels were measured in hFob1.19 cells. All data are presented as the mean ± SD of three independent experiments. *P < 0.05, **P < 0.01, ***P < 0.001, ****P < 0.0001

The relationship between HG levels and autophagy has been studied in a variety of diseases, and it has been reported that HG induces autophagy in MC3T3-E1 osteoblasts (Zhang et al. 2020a, b). In this study, we first examined autophagic flux using an autophagy double-labeled adenovirus (mRFP-GFP-LC3), which was based on the different pH stabilities of the fluorescent proteins GFP and mRFP. The results showed that hFob1.19 cells treated with HG (26.25 mM, 35 mM, and 43.75 mM) formed both yellow (representing autophagosomes) and red (representing autolysosomes) puncta, which were significantly increased compared with those in the NG group (Fig. 3A). Beclin 1 and Atg5, which are autophagy-associated proteins, participate in different stages of autophagy and play crucial roles in regulating autophagy. Western blot analysis showed that Beclin 1 and Atg5 protein expression were increased in glucose-treated hFob1.19 cells in a dose-dependent manner (Fig. 3B). To further detect autophagy levels in hFob1.19 cells, we assessed microtubule-associated protein light chain 3-II (LC3-II) conversion, and found that HG treatment resulted in a marked increase in the LC3II/I ratio in hFob1.19 cells (Fig. 3C).

Ceramide is a central molecule in the sphingolipid metabolic pathway and is involved in a variety of cellular activities, including cell proliferation, apoptosis, and autophagy (Pani et al. 2021; Sheridan and Ogretmen 2021; Zalewska et al. 2019). It has also been reported that ceramide accumulates during Era-induced ferroptosis (Thayyullathil et al. 2021). Ceramide can be generated through the hydrolysis of sphingomyelin, which involves the activation of ASM. In this study, we found that ASM expression and ceramide levels increased in hFob1.19 cells in response to HG treatment (Fig. 3B, D). Therefore, we hypothesize that ASM-mediated ceramide accumulation may be involved in the process of HG-induced ferroptosis.

### ASM is required for HG-induced ferroptosis

**Fig. 4:**
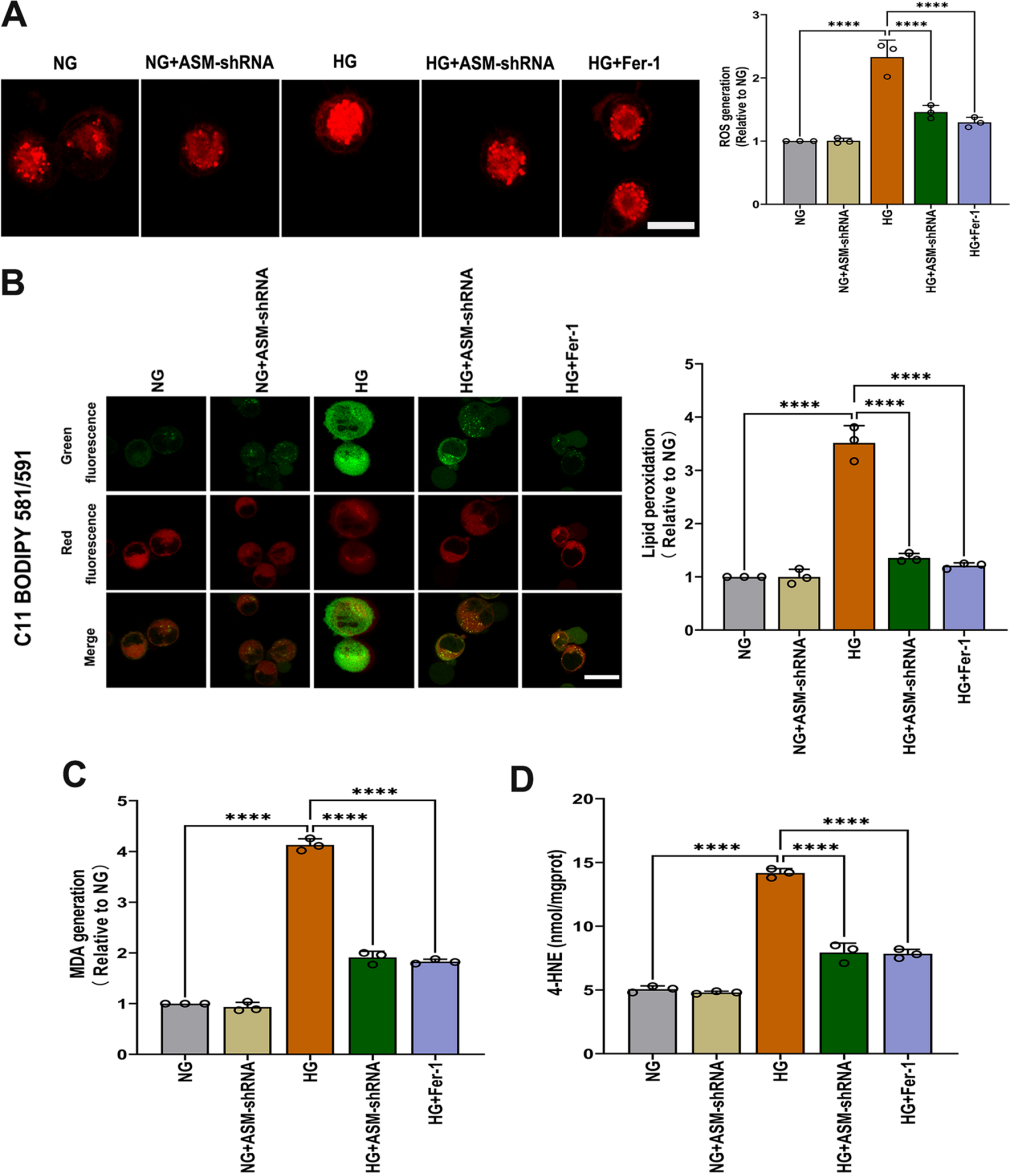
ASM is involved in HG-induced ferroptosis. ASM-shRNA-transfected hFob1.19 cells were treated with NG (17.5 mM) or HG (35 mM) for 48 h. hFob1.19 cells were treated with HG in the presence or absence of Fer-1 (5 µM). Following the treatment, **A** ROS generation was demonstrated by the DHE assay kits. **B** LPO was detected using C11 BODIPY 581/591 fluorescent probe. Scale bar = 20 μm. **C** LPO by MDA assay was determined. **D** LPO was detected by the 4-HNE assay kits. All data are presented as the mean ± SD of three independent experiments. *P < 0.05, **P < 0.01, ***P < 0.001, ****P < 0.0001

As we have confirmed, treating hFob1.19 cells with HG significantly activated ASM (Fig. 3B). Subsequently, to verify that ASM is required for HG-induced ferroptosis, an shRNA approach was used. We found that shRNA-mediated knockdown of ASM significantly reduced the accumulation of ROS (Fig. 4A) and lipid peroxides (Fig. 4B–D) induced by HG, which was consistent with the effect of the ferroptosis inhibitor Fer-1, indicating that ASM plays a crucial role in ferroptosis induced by HG.

### The role of ASM in the viability and osteogenic function of osteoblasts under HG conditions

**Fig. 5:**
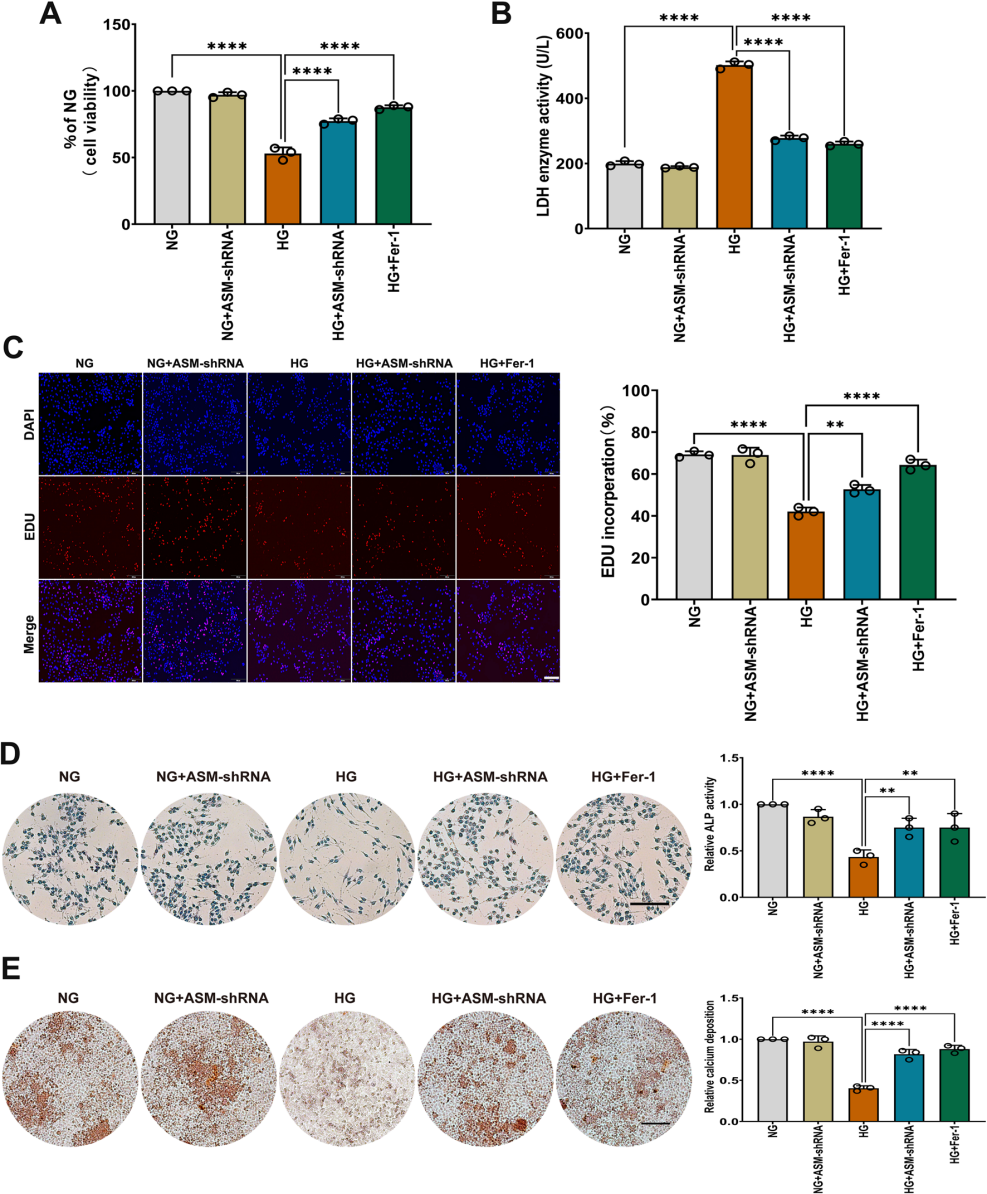
ASM decreases osteogenic potential by inducing ferroptosis in hFob1.19 cells. hFob1.19 cells were treated with NG (17.5 mM) or HG (35 mM) for 48 h in the presence or absence of ASM-shRNA. The cells were cultured with Fer-1 (5 µM) followed by HG for 48 h. Following the treatment, **A** cell survival was tested by CCK-8 assay. **B** Cell cytotoxicity was quantified using LDH content kit. **C** The proliferation of hFob1.19 cells was detected by EdU assay. Scale bar = 200 μm. **D** The osteogenic function of hFob1.19 cells was evaluated using an ALP staining kit after indicated treatments. **E** Mineralized extracellular matrix in hFob1.19cells was shown by alizarin red S staining. Scale bar = 200 μm. All data are presented as the mean ± SD of three independent experiments. **P < 0.01, ****P < 0.0001

Based on these results, we concluded that knocking down ASM could attenuate ferroptosis. In addition, our data showed that knocking down ASM increased cell viability (Fig. 5A), decreased LDH enzyme activity (Fig. 5B), enhanced cell proliferation (Fig. 5C), increased ALP activity (Fig. 5D), and increased mineralized nodule formation (Fig. 5E), indicating that knocking down ASM improved the viability and osteogenic function of hFob1.19 cells. Similarly, we used a ferroptosis inhibitor (Fer-1) to achieve the same outcomes. These results suggested that the downregulation of ASM reduced LPO and inhibited ferroptosis, thereby improving the cell viability and the osteogenic function of osteoblasts under HG conditions.

### ASM-mediated activation of autophagy is required for HG-induced ferroptosis

**Fig. 6:**
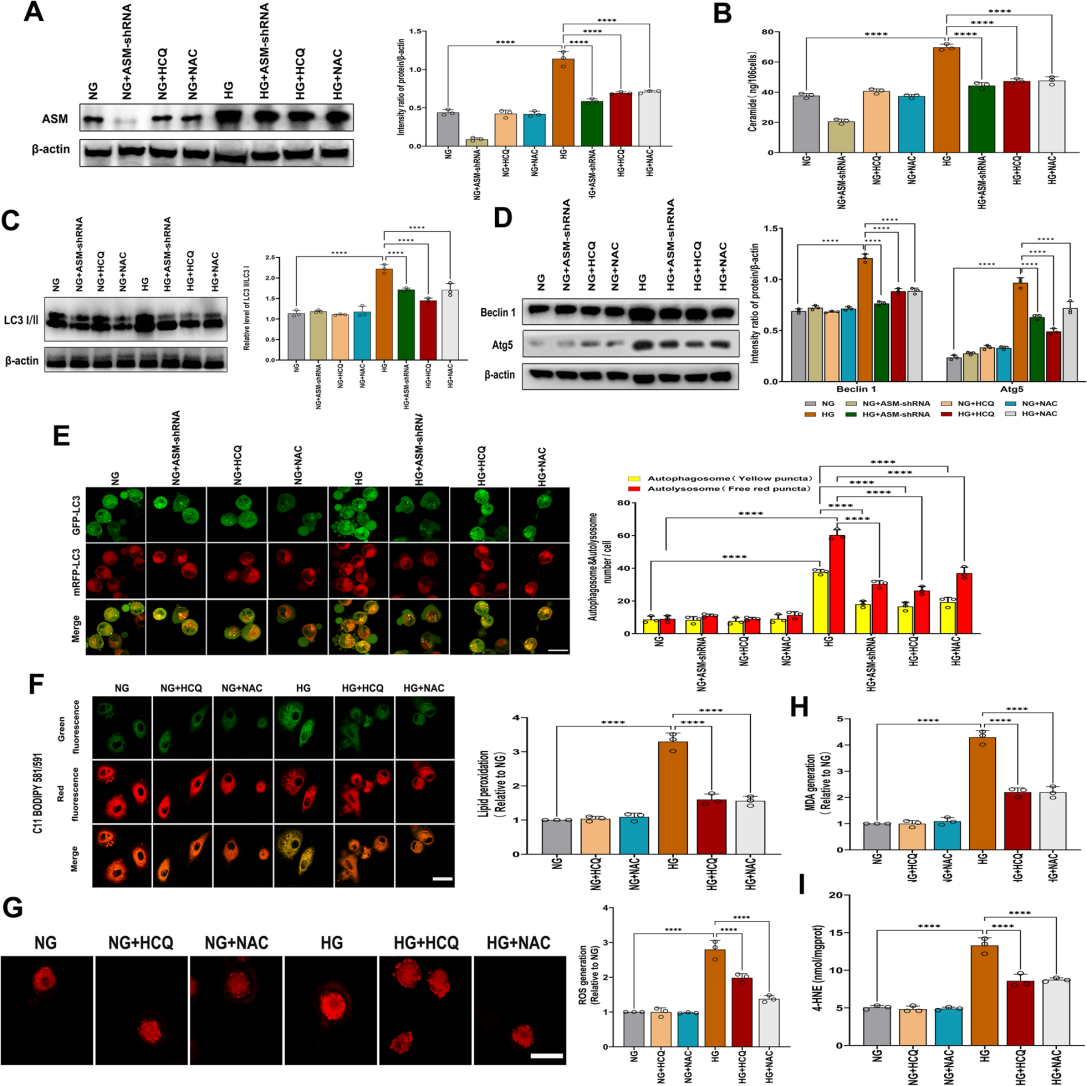
Pivotal role of ASM in the regulation of HG-induced autophagy for the execution of ferroptosis. hFob1.19 cells were treated with HG (35 mM) in the presence or absence of ASM-shRNA, HCQ (10 µM) and NAC (5 mM). Following the treatment, **A** ASM expression was determined using Western blot analysis. **B** Ceramide levels were measured. **C** Western blot analysis of LC3 II was carried out. **D** Western blot analysis of indicated proteins were carried out. **E** Autophagic flux was determined by ptfLC3 fluorescent probe. Scale bar = 20 μm. **F** LPO by C11 BODIPY 581/591 fluorescent probe was assessed. Scale bar = 20 μm. **G** ROS generation was detected by DHE assay kits. Scale bar = 20 μm. **H** LPO was determined by MDA assay. **I** LPO was detected by the 4-HNE assay kits. All data are presented as the mean ± SD of three independent experiments.****P < 0.0001

To verify whether ASM is involved in HG-induced autophagy, we measured LC3-II conversion, Beclin 1 and Atg5 expression and autolysosome formation in ASM-knockdown cells under HG conditions. The results showed that inhibiting ASM activity with ASM-shRNA decreased ceramide production (Fig. 6A, B), significantly alleviated the HG-induced upregulation of LC3-II, Beclin 1, and Atg5 (Fig. 6C, D), and inhibited both yellow (representing autophagosomes) and red puncta (representing autolysosomes) formation induced by HG (Fig. 6E), suggesting that ASM-dependent ceramide generation was critical for HG-induced autophagy.

Autophagy is a critical regulator of cellular homeostasis and participates in various physiological and pathological conditions. In many cases, excessive or uncontrolled levels of autophagy can trigger autophagy-dependent cell death. Recently, several studies have shown that ferroptosis is accompanied by the activation of autophagy (Zhou et al. 2020). Next, we determined the production of lipid peroxide and ROS in hFob1.19 cells treated with HCQ and found that HG-induced lipid peroxide and ROS accumulation were significantly inhibited by HCQ treatment (Fig. 6F–I). These results showed that ASM-mediated autophagy could play an important role in facilitating autophagy-mediated ferroptosis.

### ASM-mediated ROS accumulation amplifies HG-induced ASM activation

The data presented above indicate that HG induces ROS accumulation in hFob1.19 cells (Fig. 1F), and ROS release is significantly inhibited in cells treated with ASM-shRNA (Fig. 4A). Redox regulation of ASM has been confirmed in Pseudomonas aeruginosa-induced macrophage apoptosis (Zhang et al. 2008), and we examined whether there was also positive feedback regulation between ASM activation and ROS generation during HG-induced ferroptosis. As shown in Fig. 6A HG-induced ASM activation was significantly inhibited by treatment with the ROS inhibitor NAC. Similarly, the accumulation of lipid peroxides and ROS induced by HG was also significantly inhibited by NAC treatment (Fig. 6F–I). Therefore, these results indicate that ASM and ROS production regulate each other in a positive feedback manner during HG-induced ferroptosis.

## ASM is required for the autophagic degradation of GPX4 in HG-induced ferroptosis

**Fig. 7:**
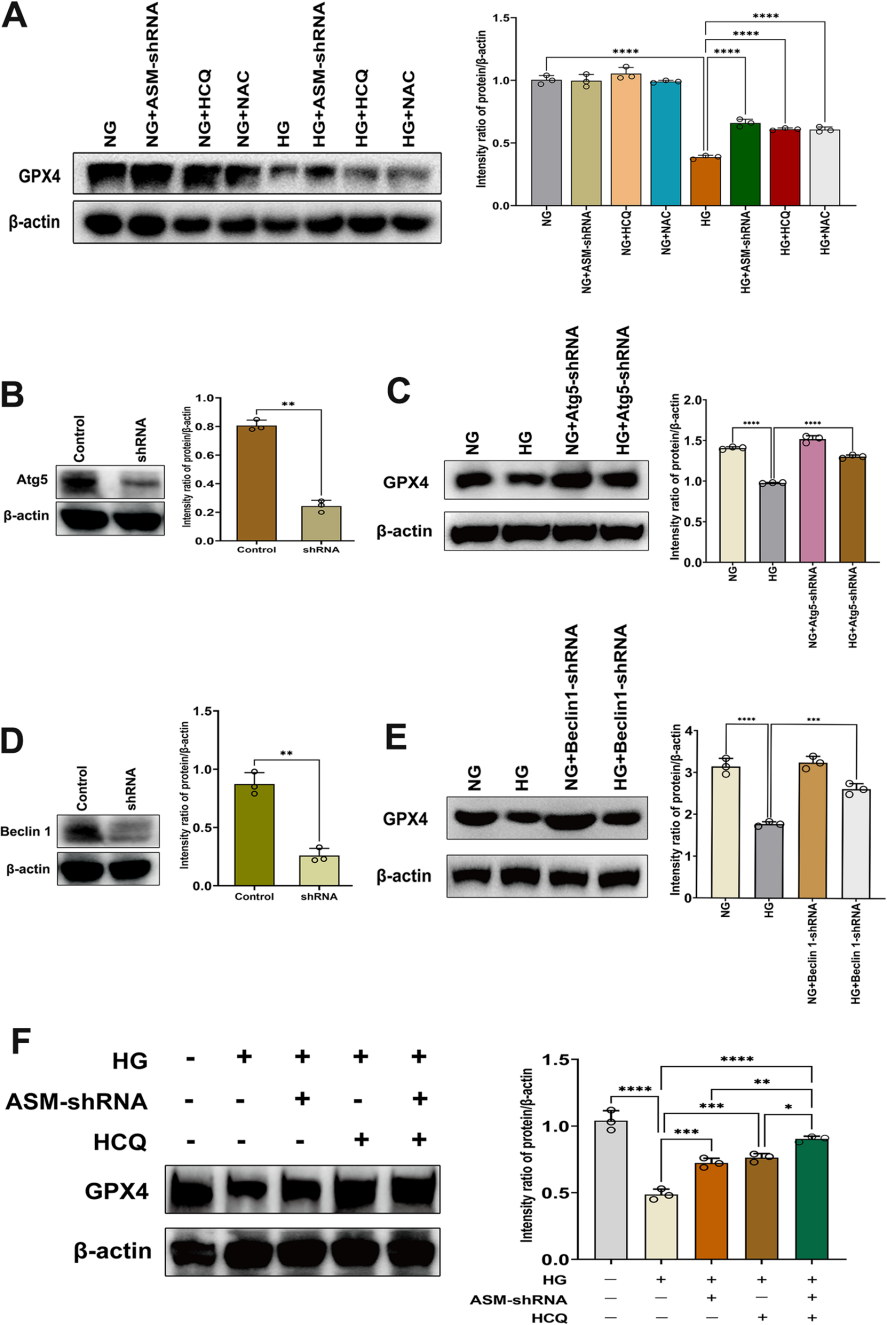
Contribution of ASM to the autophagic degradation of GPX4. hFob1.19 cells were treated with HG (35 mM) in the presence or absence of ASM-shRNA, HCQ (10 µM) and NAC (5 mM). Following the treatment, **A** Western blot analysis of GPX4 expression was carried out. Relative density of each protein bands were quantified, and the data are presented as the mean ± SD of three independent experiments. ****P < 0.0001. **B** Atg5 protein levels inhFob1.19 cells demonstrated by Western blotting. **C** hFob1.19 cells were treated with HG (35 mM) in the presence or absence of Atg5-shRNA. Western blot analysis of GPX4 was carried out. **D** Beclin 1 protein levels in hFob1.19 cells verified by Western blot analysis. **E** hFob1.19 cells were treated with HG (35 mM) in the presence or absence of Beclin 1-shRNA. Western blot analysis of GPX4 was carried out. **F** ASM-shRNA-transfected hFob1.19 cells were treated with HCQ (10 µM) for 12 h. Western blot analysis of GPX4 was carried out. All data are presented as the mean ± SD of three independent experiments. *P < 0.05, **P < 0.01,***P < 0.001, ****P < 0.0001

Although the anti-ferroptotic function of GPX4 has been extensively studied, the regulatory mechanism of GPX4 remains poorly understood. It has been reported that the degradation of GPX4 is crucial for the accumulation of lipid peroxides in many instances of ferroptosis (Seibt et al. 2019; Lei et al. 2019), and autophagy has recently been reported to be involved in GPX4 degradation (Xue et al. 2023; Chen et al. 2021). As we observed, HG dose-dependently reduced GPX4 expression in hFob1.19 cells (Fig. 1G). To determine the direct association between ASM activation and ferroptosis, we examined whether inhibiting ASM expression could suppress GPX4 degradation. As expected, genetic inhibition of ASM with shRNA significantly decreased HG-induced GPX4 degradation, suggesting that the inhibition of ASM antagonizes ferroptosis by preserving physiological levels of active GPX4 (Fig. 7A). To further explore how ASM regulates the degradation of GPX4, we treated hFob1.19 cells with the autophagy inhibitors HCQ prior to HG treatment and found that HCQ protected against GPX4 degradation (Fig. 7A). Moreover, we used shRNA approach to knock down Atg5 (Fig. 7B) and Beclin1 (Fig. 7D), respectively, and found that genetic inhibition of autophagy also inhibited GPX4 degradation (Fig. 7C, E). To further determine whether ASM regulates GPX4 degradation in an autophagy-dependent manner, ASM-shRNA-transfected hFob1.19 cells were treated with HCQ. Interestingly, the autophagy inhibitor amplified the inhibition of GPX4 degradation mediated by ASM-shRNA (Fig. 7F). Since inhibiting of ASM reduced ROS production and vice versa (Figs. 4A, 6A), we checked whether inhibiting ROS production could protect against HG-induced autophagy and GPX4 degradation. Interestingly, the ROS inhibitor NAC prevented HG-induced autophagy (Fig. 6C–E) and GPX4 degradation (Fig. 7A). Taken together, these results indicate that ASM-mediated redox amplification regulates the autophagic degradation of GPX4, leading to ferroptosis in hFob1.19 cells and decreasing in osteoblast viability and osteogenic function.

### Establishment of the T2DOP rat model

**Fig. 8:**
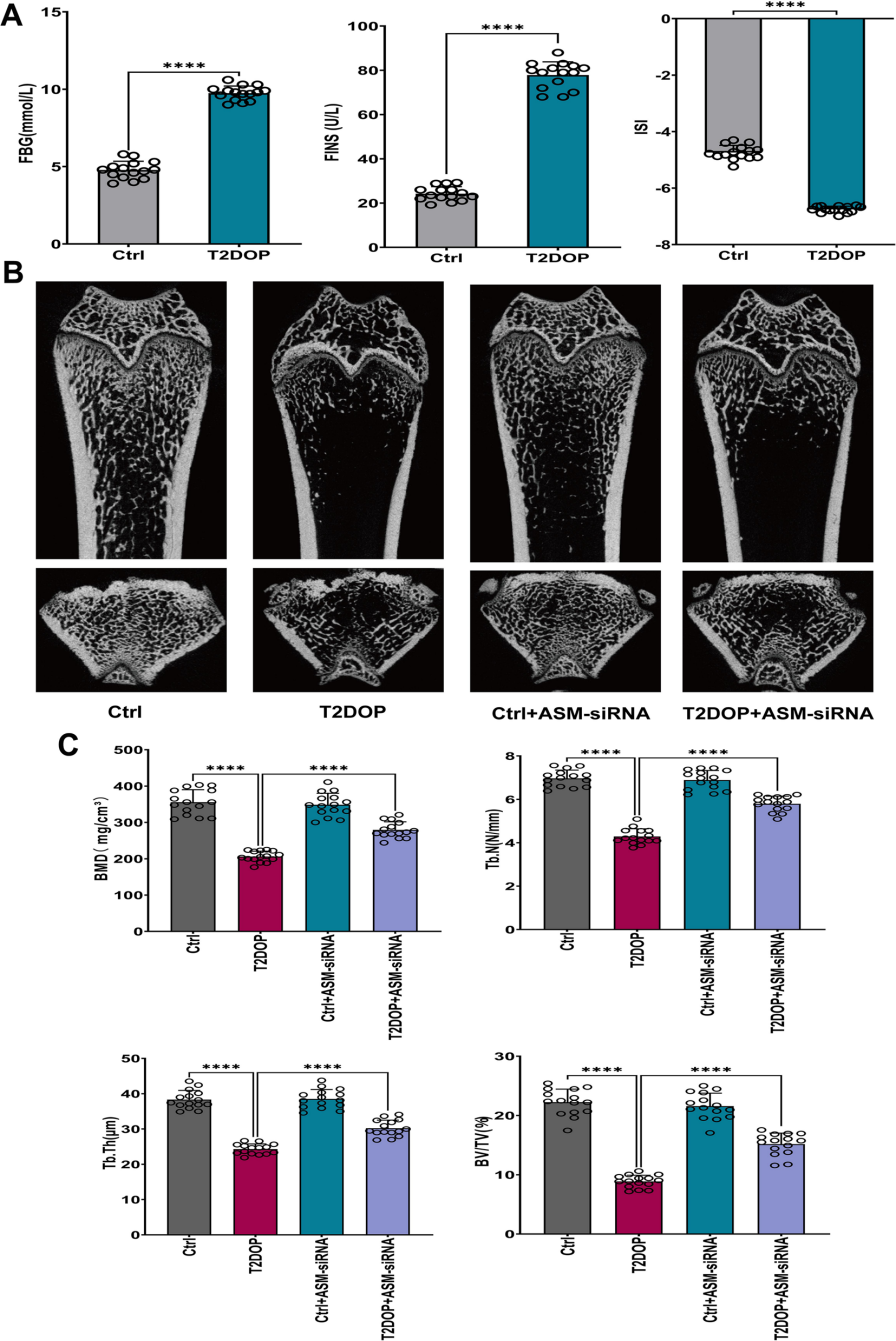
Establishment of the T2DOP rat model. **A** FBG and FINS increased and ISI decreased in T2DOP rats. ISI= − ln(FBG × FINS). **B** Micro-CT analysis of the distal metaphyseal femur region. **C** Micro-CT-based quantification of BMD, Tb.N, Tb.Th and BV/TV. N = 15, ****P < 0.0001

The rat model was induced by high-fat feeding combined with intraperitoneal injection of streptozotocin, and the successful establishment of the T2DM rat model was evaluated by measuring fasting blood glucose (FBG), fasting insulin (FINS), and insulin sensitivity index (ISI) (Fig. 8A). Then, we assessed bone microstructure using micro-CT to determine the establishment of the T2DOP model. The results showed that BMD, BV/TV, Tb.N, and Tb.Th were significantly reduced in the experimental group rats (Fig. 8B, C), suggesting that bone quality was significantly worse in the rat model of T2DOP.

### The effect of ASM on T2DOP rats

**Fig. 9:**
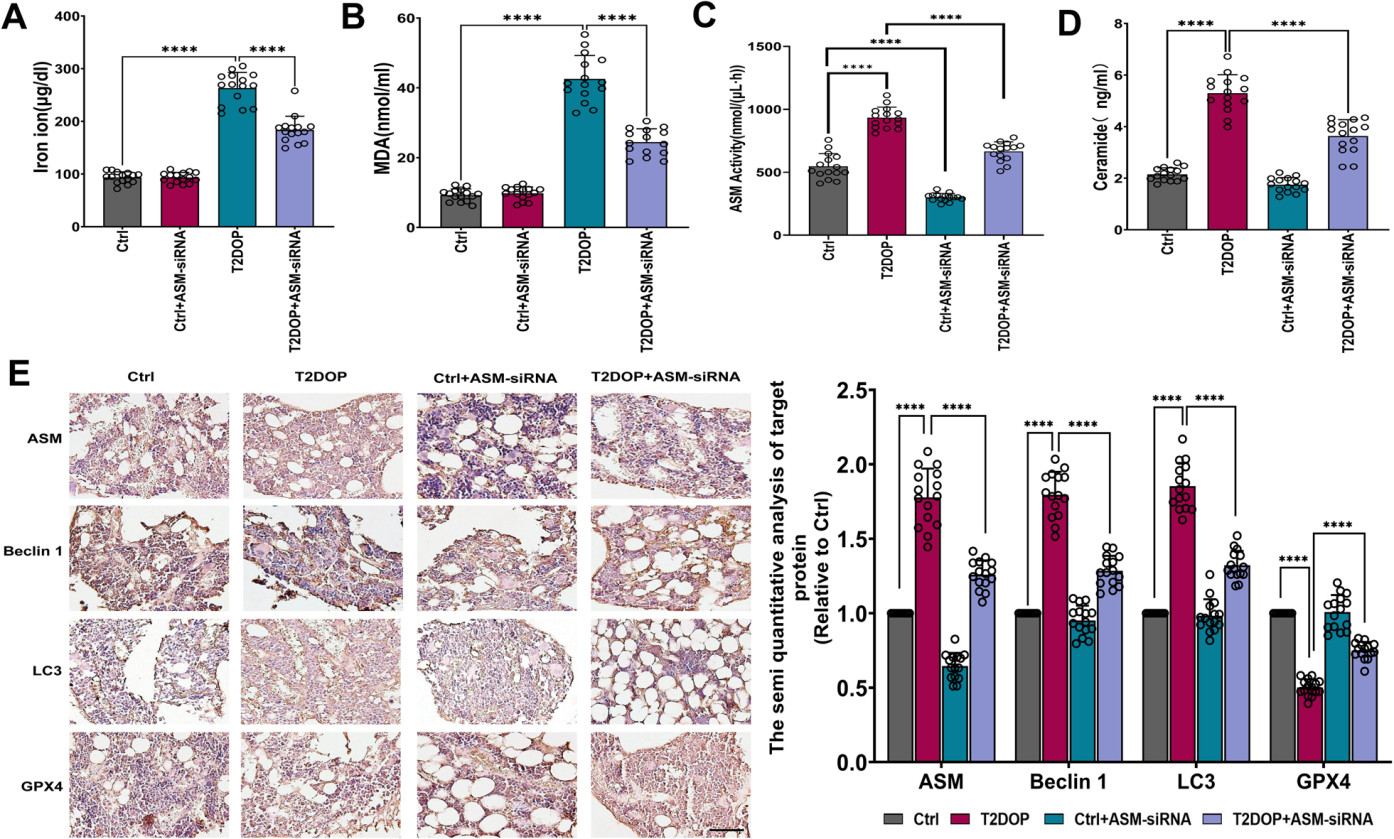
Effect and potential mechanism of ASM on T2DOP. Sixty SD rats were divided into four groups (n = 15). **A** Determination of iron ion levels in serum. **B** MDA measurement in serum. **C** Determination of ASM activity in serum. **D** Detection of ceramide in serum. **E** IHC for ASM, Beclin 1, LC3 and GPX4 in four groups of rats. Scale bar = 100 μm. ****P < 0.0001

We found that it was difficult to detect iron ions and MDA in bone tissue after some attempts, so we tested the relevant indicators in the serum. Consistent with previous reports, the serum levels of iron ions (Fig. 9A) and MDA (Fig. 9B) were increased in T2DOP rats. Moreover, ASM activity and ceramide levels were increased in T2DOP rats (Fig. 9C, D). IHC analysis showed decreased expression of GPX4 in the bone tissue of T2DOP rats (Fig. 9E), indicating that ferroptosis was involved in the pathogenesis of T2DOP. Thus, inhibiting ferroptosis may significantly improve T2DOP.

Next, we knocked down ASM in T2DOP rats using ASM-siRNA. In T2DOP rats with ASM knockdown, serum levels of iron ions, MDA and ceramide (Fig. 9A, B, D), as well as ASM activity (Fig. 9C), were decreased. The IHC data showed that GPX4 expression levels were significantly increased compared to those in T2DOP rats, suggesting an improvement in ferroptosis (Fig. 9E). In addition, ASM knockdown increased the values of BMD, Tb.N, Tb.Th and BV/TV (Fig. 8B, C), demonstrating that inhibiting the function of ASM could effectively improve ferroptosis and reduce the severity of T2DOP.

## Discussion

Diabetes mellitus (DM) typically leads to osteoporosis, which is often associated with multiple factors. It has been reported that the factors contributing to the reduction in bone formation include oxidative stress andiron overload caused by HG (Manolagas 2010; Wang et al. 2022).The recently identified form of programmed cell death known as ferroptosis is generally considered to be a nonapoptotic cell death pathway driven by iron-mediated production of ROS and subsequent LPO (Su et al. 2019). In recent years, ferroptosis has been reported to be involved in the pathogenesis of T2DOP, although its specific mechanism has not been fully clarified (Zhang et al. 2022; Gao et al. 2022). In our previous study, we found that the accumulation of ROS and lipid peroxides and the inhibitory effect on osteoblast survival and osteogenesis caused by HG could be reversed by treatment with the ferroptosis inhibitor Fer-1, suggesting that ferroptosis was induced by HG in osteoblasts (Zhao et al. 2022). The present study further confirmed this point.

Lipids have long been recognized as contributors to the pathogenesis and pathophysiology of DM and its complications (Tabak et al. 2011). Sphingolipid metabolism is a prominent lipid metabolic pathway in cells, and ASM is one of its main components. ASM can hydrolyze sphingomyelin to produce ceramide and prevent its accumulation in cells. Recent discoveries have highlighted ceramide, which is a central molecule in sphingolipid metabolism, as a potential driving force in many pathologic conditions, including DM and DM-associated complications (Mandal et al. 2021).

The ASM/ceramide system plays established cellular signaling roles in a diverse array of stresses, such as heat shock, UV irradiation, and oxidative stress (Li et al. 2012; Kornhuber et al. 2011). The excessive activation of ASM and ceramide production induced by sphingolipid metabolic abnormalities and dysregulation can occur in response to environmental insults, infection with pathogens, ligation of death receptors, drugs, and other stressors. ASM not only plays a fundamental role in apoptotic cell death by participating in several pathophysiological conditions but also promotes mitochondrial dysfunction in glutamate-induced ferroptosis in oligodendrocytes (Novgorodov et al. 2018). Recently, it has been reported that ASM contributes to the core molecular machinery and signaling pathways involved in ferroptosis induced by erastin in HT1080 cells (Thayyullathil et al. 2021). ASM deficiency is caused by autosomal recessive mutations in SMPD1, and this disease is known as Niemann–Pick diseases in human. However, these patients do not seem to have bone problems. In this study, we similarly found that inhibiting ASM expression in control rats did not affect bone mass, indicating that ASM deficiency is not a risk factor for osteoporosis. These results demonstrate that HG triggers selective activation of ASM, leading to significant generation of ceramide in hFob1.19 cells. We showed that the kinetics of ASM activation, ceramide generation, and ferroptosis parallel each other, suggesting the critical involvement of the ASM/ceramide pathway in the ferroptosis process.

As an evolutionarily conserved lysosomal degradative pathway, autophagy plays dual roles in preventing or promoting death and is executed by a set of autophagy-related proteins. Although autophagy-related stress tolerance can enable cells to survive under various types of cellular death stimuli, uncontrolled or excessive autophagy levels restrict autophagy-dependent cell death (Kriel and Loos 2019). In this study, we evaluated the levels of autophagy in hFob1.19 cells by examining autophagic markers and autophagic flux. The autophagy inhibitor HCQ significantly inhibited HG-induced LC3-II accumulation (autophagic flux) and autophagic markers expression (Beclin 1 and Atg5). Interestingly, the autophagy inhibitor also significantly inhibited HG-induced ROS generation, LPO and cell death. As stated in the literature, some types of selective autophagy contribute to ferroptotic cell death. For instance, NCOA4-mediated ferritinophagy, RAB7A-dependent lipophagy, and STAT3-induced lysosomal membrane permeabilization can promote ferroptosis (Del Quiles and Mancias 2019; Lin et al. 2022; Zhang et al. 2020a, b). The present study indicates a close relationship between autophagy and ferroptosis.

The function of ASM in autophagosome formation is well established (Perrotta et al. 2015). To determine whether ASM regulates ferroptosis in an autophagy-dependent manner, we measured Beclin 1 and Atg5 expression and autophagic flux in ASM-knockdown cells in the presence or absence of HG. Inhibiting ASM activity by knocking down ASM expression using shRNA significantly inhibited HG-induced autophagy, indicating that ASM plays an important role in facilitating autophagy-mediated ferroptosis. Overall, these findings indicate that ASM-mediated autophagy is essential for the facilitation of ferroptosis.

GPX4 is a central defense enzyme against LPO and plays a unique role in the inhibition of ferroptosis (Ursini and Maiorino 2020). Its degradation or inactivation is an indispensable signaling event in the process of ferroptosis (Sun et al. 2021). In this study, we demonstrated a distinct mechanism through which ASM induces GPX4 degradation in response to ferroptosis activators. ROS accumulation is one of the indispensable hallmarks of ferroptosis, and ROS have been reported to oxidize the C-terminal Cys629 residue of ASM, leading to its activation (Qiu et al. 2003). In our study, we demonstrated that HG induced ROS accumulation, which was significantly inhibited by knocking down ASM expression. Meanwhile, the antioxidant NAC significantly inhibited HG-induced ASM activation. Therefore, these results point to a positive cyclic correlation between ASM activation and ROS generation in the facilitation of HG-induced ferroptosis. Mechanistically, we revealed that ASM was required for autophagic degradation of GPX4 by enhancing HG-induced ROS production. Inhibiting ASM decreased HG-induced ROS production and autophagy, which led to the inhibition of GPX4 degradation and LPO and an increase in cell survival. Unfortunately, we did not study the de novo transcription of GPX4 and its influence on other GPX4 degradation pathways in response to HG. Due to other pathways involved in this process, such as GSH depletion and GPX4 inactivation. Therefore, the autophagic degradation of GPX4 described here cannot be determined to be the predominant way to maintain GPX4 levels.

It has been demonstrated that HG conditions in T2DM severely weaken the biological functions of osteoblasts, which is caused by the accumulation of ROS and lipid peroxides (Ma et al. 2020). HG conditions also affect osteoblasts by modulating iron metabolism. It has been reported that iron metabolism can affect bone homeostasis through ferroptosis, which is a kind of iron-dependent cell death characterized by the accumulation of lipid peroxides and ROS (Miao et al. 2023). In the present study, we found increased serum levels of iron ions and MDA in T2DOP rats, which were negatively correlated with the expression of GPX4, indicating that ferroptosis occurred in T2DOP rats. In T2DOP rats with ASM knockdown, iron metabolism and LPO levels were significantly reduced, resulting in an improvement in the severity of osteoporosis. In addition, we verified that the autophagy level in T2DOP rats was significantly increased and decreased in response to ASM knockdown. As expected, these animal experimental results are consistent with the in vitro cell experiments. Unfortunately, we have not yet attempted to treat other cell types in HG to test the specificity of this pathway in bone cells.

## Conclusions

In summary, we identified a regulatory signaling pathway mediated by ASM that positively controls HG-induced osteoblast ferroptosis by activating the autophagic degradation of GPX4. ASM-mediated autophagy-dependent ferroptosis may be a potential target for the development of pharmacological agents to enhance or inhibit ferroptosis signaling pathways. Importantly, ASM may be a key regulator of osteoblastic ferroptosis in the T2DOP rat model, providing a new target for the treatment and prevention of T2DOP.

## Data Availability

The data and materials of the study can be obtained from the corresponding author upon request.
